# Beyond Lipids and Platelets: A Review of Anti-Inflammatory Strategies in Secondary Prevention of Acute Coronary Syndromes

**DOI:** 10.3390/jcm14227910

**Published:** 2025-11-07

**Authors:** Girish Pathangey, Mohamed N. Allam, Mahmoud H. Abdelnabi, Dan Sorajja, Floyd David Fortuin, Kwan S. Lee, Mayurkumar D. Bhakta

**Affiliations:** Department of Cardiovascular Medicine, Mayo Clinic, Phoenix, AZ 85054, USA; allam.mohamed@mayo.edu (M.N.A.); abdelnabi.mahmoud@mayo.edu (M.H.A.); fortuin.david@mayo.edu (F.D.F.); lee.kwan@mayo.edu (K.S.L.); bhakta.mayurkumar@mayo.edu (M.D.B.)

**Keywords:** acute coronary syndromes, atherosclerosis, plaque inflammation, residual risk, biomarkers, hsCRP, IL-6, NLRP3 inflammasome, immunomodulation, anti-inflammatory therapy, cardiometabolic therapies, GLP-1 receptor agonists, SGLT2 inhibitors, precision medicine, secondary prevention, acute myocardial infarction, coronary artery disease, translational research, novel therapeutics

## Abstract

Despite advances in lipid-lowering and antithrombotic therapy, patients with acute coronary syndromes remain at elevated risk for recurrent events due to persistent atherosclerotic inflammation. This review evaluates inflammation as a therapeutic target in secondary prevention and discusses established, investigational, and emerging strategies. Colchicine, now FDA-approved for cardiovascular risk reduction, lowered major adverse cardiovascular events in COLCOT and LoDoCo2. Canakinumab (IL-1β inhibition) reduced recurrent events in proportion to IL-6 and hsCRP suppression, while ziltivekimab (IL-6 inhibition) achieved profound biomarker reductions but remains investigational. Early-phase studies of anakinra (IL-1 receptor antagonist) and dapansutrile (oral NLRP3 inhibitor) showed anti-inflammatory effects in early trials, whereas varespladib and darapladib illustrated the challenges of targeting lipid-associated pathways. Beyond direct immunomodulators, GLP-1 receptor agonists and SGLT2 inhibitors provide both cardioprotective and anti-inflammatory benefits, reinforcing their expanding role post-ACS. Additional emerging avenues include triptolidiol, dasatinib, and BTK or JAK/STAT inhibitors, while novel approaches, such as nanozyme delivery systems and CRISPR-based editing, extend the therapeutic horizon. This review highlights the potential of inflammation-targeted therapies to advance secondary prevention in ACS by integrating current evidence and perspectives on future therapeutic developments.

## 1. Introduction

Inflammation is integral to the initiation, progression, and destabilization of atherosclerotic plaques that lead to acute coronary syndromes (ACS) [1,2,3]. Despite contemporary lipid-lowering and antithrombotic strategies, a significant proportion of patients continue to experience recurrent ischemic events driven by residual inflammatory risk (RIR) [4]. High-sensitivity C-reactive protein (hsCRP) and interleukin-6 (IL-6) are well-established biomarkers of RIR, with elevated levels independently associated with adverse cardiovascular outcomes even when low-density lipoprotein cholesterol (LDL-C) is optimally controlled. These observations have catalyzed a paradigm shift towards inflammation-targeted strategies as adjuncts in secondary prevention [5,6,7].

Randomized trials and meta-analyses over the past decade have demonstrated that targeted anti-inflammatory therapy has the potential to reduce major adverse cardiovascular events (MACE) in high-risk populations. Low-dose colchicine provides the strongest evidence base amongst anti-inflammatory therapies across post-myocardial infarction (MI) and chronic coronary disease [8,9,10]. The currently investigated cytokine-directed approaches offer mechanistic proof of concept, with interleukin-1β inhibition (IL-1β) lowering events independent of lipid lowering and IL-6 inhibition producing significant biomarker reductions pending outcome confirmation. Cardiometabolic agents, such as glucagon-like peptide-1 receptor agonists (GLP-1 RAs) and sodium-glucose cotransporter-2 inhibitors (SGLT2i), provide complementary metabolic and anti-inflammatory benefits, whereas emerging strategies aim upstream by targeting NLRP3 activation, modulating epigenetic and transcriptional programs, and restoring vascular immune homeostasis to achieve precision control of post-ACS inflammation [11,12,13,14,15].

This review examines the current evidence on validated, investigational, and emerging anti-inflammatory therapies following ACS (graphical abstract), evaluates safety and practical implementation, and outlines key directions for precise secondary prevention.

## 2. Pathophysiology of Inflammation in ACS

**Figure 1:**
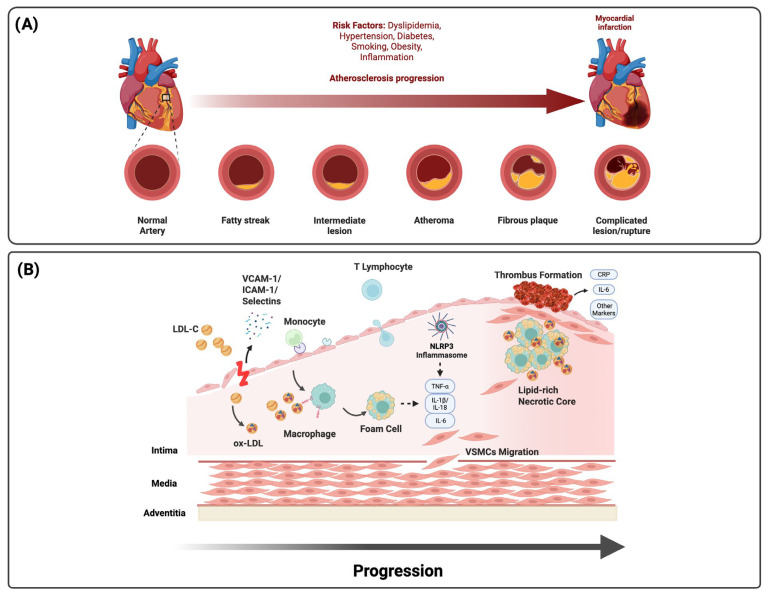
Pathophysiology of atherosclerosis and inflammation in ACS. (**A**) Atherosclerotic progression from fatty streaks to complicated lesions and rupture is accelerated by dyslipidemia, diabetes, hypertension, obesity, smoking, and systemic inflammation. (**B**) Endothelial activation and LDL infiltration drive monocyte recruitment, foam cell formation, and necrotic core expansion. NLRP3 inflammasome activation amplifies cytokine release (IL-1β, IL-18, IL-6, and TNF-α), promoting plaque destabilization, thrombosis, and systemic residual inflammatory risk reflected by circulating biomarkers (CRP and IL-6).

ACS, comprising ST-elevation myocardial infarction (STEMI), non-ST-elevation myocardial infarction (NSTEMI), and unstable angina, represents an acute manifestation of chronic atherosclerotic disease driven by endothelial dysfunction and arterial wall inflammation in response to cardiovascular risk factors such as hyperlipidemia, hypertension, diabetes mellitus, and smoking [1,2,3,4,7]. Inflammation drives every stage of atherosclerosis, from lesion initiation and progression to destabilization, rupture, and thrombosis, with both innate and adaptive immune responses influencing clinical presentation, recurrence risk, and therapeutic outcomes [5,7,16]. Systemic inflammatory states, including autoimmune disease, chronic infection, obesity, and metabolic dysregulation, further prime vascular and circulating immune cells toward a pro-atherogenic phenotype via persistent cytokine activation, lowering the threshold for acute events (Figure 1).

### 2.1. Initiation and Propagation of Atheroinflammation

Endothelial activation initiates the inflammatory cascade that promotes leukocyte adhesion and transendothelial migration. Selectins and adhesion molecules (VCAM-1 and ICAM-1) mediate rolling and firm attachment through integrins VLA-4 (α4β1) and LFA-1 (αLβ2) [16,17]. Within this adhesive interface, chemokine gradients direct immune recruitment, with monocyte trafficking governed by the CCL2-CCR2, CX3CL1-CX3CR1, and CCL5-CCR5 axes and neutrophil migration mediated by CXCL1-CXCR2 (Table 1) [3,17]. These pathways are modifiable; intensive lipid lowering reduces CCR2-dependent monocyte mobilization and endothelial activation, linking metabolic therapy to vascular immune control [18,19]. Once within the intima, monocytes differentiate into macrophages that internalize oxidized LDL, form foam cells, and initiate atherosclerotic plaque formation.

Macrophage polarization governs plaque evolution and stability. M1-like macrophages activated by TLR ligands and IFN-γ secrete TNF-α, IL-1β, and matrix metalloproteinases that degrade collagen, thin the fibrous cap, and expand the necrotic core. In contrast, M2-like macrophages stimulated by IL-10 and TGF-β enhance efferocytosis, promote collagen synthesis, and facilitate resolution [3,23]. Impaired efferocytosis allows apoptotic cells to undergo secondary necrosis, sustaining inflammation and enlarging the lipid core. These macrophage programs shape plaque phenotype, predisposing it to rupture via cap thinning or to erosion driven by endothelial TLR2 activation and oxidative stress [24,25,26].

The NLRP3 inflammasome links metabolic stress to innate immune amplification. Priming through TLR2/4 or RAGE engagement by oxidized LDL and other damage-associated molecular patterns induces NLRP3 and pro-IL-1β transcription, while activation by cholesterol crystals, ATP-P2X7-mediated potassium efflux, mitochondrial ROS or DNA release, hyperglycemia, or ischemia–reperfusion triggers caspase-1, producing IL-1β and IL-18 [16,20]. This cascade amplifies cytokine signaling and triggers gasdermin-D-mediated pyroptosis, releasing alarmins that perpetuate vascular inflammation. The IL-1β/IL-6 axis couples local plaque activity with systemic inflammatory markers, linking innate activation to clinical outcomes [3,27].

Adaptive immune responses further refine these pathways. Th1 and Th17 subsets release IFN-γ and IL-17 to amplify macrophage activation and neutrophil recruitment, whereas regulatory T and Th2 cells counterbalance inflammation through IL-10 and TGF-β. Dendritic cells sustain chronic activation through continuous antigen presentation [3]. These pattern-recognition, chemokine, and adhesion networks define a receptor-to-fate cascade that governs macrophage polarization, fibrous-cap integrity, and transition from subclinical inflammation to plaque rupture, erosion, or impaired healing [3,17,25]. This framework provides a mechanistic rationale for targeted anti-inflammatory therapy in secondary prevention.

### 2.2. Plaque Destabilization and Thrombosis

Transition to unstable plaque occurs via rupture (55–65%), erosion (30–35%), or, less commonly, calcified nodules (2–7%) [24,25,28]. Plaque rupture typically involves lipid-rich necrotic cores with thin, inflamed fibrous caps where macrophage and T cell-derived matrix metalloproteinases (MMPs) degrade collagen and compromise cap integrity [24]. Plaque erosion, more common in younger patients and women, features endothelial apoptosis and denudation without cap rupture [25]. Calcified nodules occur more often in older adults and chronic kidney disease (CKD) and can protrude through the fibrous cap and trigger thrombosis [28]. 

Plaque disruption exposes thrombogenic substrates, initiating platelet adhesion, activation, and aggregation. Activated platelets release CD40 ligand and platelet factor 4, which engage CD40 and chemokine receptors on endothelial and immune cells to induce adhesion molecules, recruit leukocytes, and stimulate cytokine release. These interactions promote a neutrophil extracellular trap (NET) formation that concentrates tissue factor, histones, and proteases, accelerating thrombin generation and stabilizing platelet-rich thrombi [17,20,29,30]. Myeloperoxidase (MPO) on NETs augments endothelial apoptosis, oxidizes LDL, and increases tissue factor expression, a hallmark of plaque erosion [29,31]. Reactive oxygen species further diminish nitric oxide availability, sustaining endothelial dysfunction and leukocyte infiltration. Microembolization of platelet–leukocyte aggregates and NET fragments can obstruct the coronary microcirculation, increasing infarct size and sustaining systemic inflammation [3,32].

Defective efferocytosis in advanced plaque impairs apoptotic cell clearance, leading to secondary necrosis, lipid-core expansion, and persistent inflammation. This dysfunction reflects cleavage of MerTK, disruption of the Gas6-MerTK axis, and inhibitory CD47-SIRPα signaling that suppresses phagocytic uptake [3,23]. Macrophage polarization and efferocytic efficiency, which is shaped by CCL2-CCR2-driven monocyte recruitment and NLRP3-dependent IL-1β activation, govern collagen turnover and cap integrity, while matrix metalloproteinase activity and impaired smooth-muscle repair further weaken the fibrous cap [3,7,24,28]. In contrast, erosion-prone plaques exhibit heightened TLR2 activation by fragmented hyaluronan and other damage-associated ligands, triggering MyD88-NF-κB signaling, endothelial activation, and neutrophil-derived NETosis on denuded surfaces [25,26]. These lesions typically have smaller lipid cores yet intense luminal inflammation, explaining modest systemic biomarkers despite high local thrombogenicity.

Following myocardial infarction, emergency myelopoiesis and splenic remodeling imprint trained immunity in circulating monocytes through chromatin remodeling (H3K4me3) and metabolic reprogramming toward glycolysis and cholesterol biosynthesis, heightening inflammatory responsiveness and recurrence risk [33]. At the same time, impaired biosynthesis of specialized pro-resolving mediators (resolvins, lipoxins, and maresins) limits efferocytosis and fibrous-cap restoration, sustaining vascular inflammation [3,32]. Collectively, platelet activation, leukocyte recruitment, and NETosis form a self-reinforcing circuit that amplifies thrombin generation and plaque thrombosis. Upstream chemokine signaling and NLRP3 activation dictate macrophage phenotype and efferocytic competence, driving rupture through collagen loss, whereas endothelial TLR2-MyD88 signaling with neutrophil recruitment promotes erosion. Persistent trained immunity and impaired resolution maintain inflammatory readiness after the index event, linking local plaque characteristics to RIR and recurrent ACS [3,7,20,26].

### 2.3. Biomarkers and Imaging of Residual Inflammatory Risk

Residual inflammatory risk after ACS reflects sustained activation of innate immune pathways despite optimal lipid-lowering and antithrombotic therapy. Biomarkers and imaging provide complementary mechanistic and prognostic insights when interpreted in a temporal and clinical context.

hsCRP, synthesized in hepatocytes under STAT3 regulation downstream of IL-6, serves as an integrative marker of IL-6-JAK-STAT3 activity [5,12]. hsCRP typically peaks 48 h after the index event and declines in uncomplicated cases, whereas IL-6 rises earlier and amplifies the acute-phase cascade through gp130-JAK-STAT3 signaling, driving hepatic CRP and fibrinogen synthesis [27]. Other biomarkers, summarized in Table 2, include NETs and MPO and serve as markers of thrombo-inflammatory activity; NETs, generated through PAD4-dependent chromatin decondensation, form scaffolds for thrombin generation and amplify endothelial injury, while circulating NET fragments and MPO-DNA complexes indicate ongoing NETosis and oxidative stress linking innate immunity to microvascular obstruction and thrombosis [20,29,31]. MPO further catalyzes LDL oxidation and promotes endothelial apoptosis, mechanisms particularly relevant in plaque erosion. Additional systemic biomarkers, such as serum amyloid A, fibrinogen, and growth-differentiation factor-15, together with leukocyte-derived ratios (NLR, MLR), integrate inflammatory and prothrombotic activity [32,34]. Emerging candidates, including GlycA, soluble uPAR, and clonal hematopoiesis (CHIP) mutations, extend mechanistic profiling but remain investigational.

hsCRP and IL-6 are the most validated markers of RIR. Elevated baseline or sustained post-ACS hsCRP and IL-6 levels predict ASCVD with strength comparable to LDL-C or Lp(a). Individuals with both hsCRP ≥ 2 mg/L and LDL-C ≥ 130 mg/dL observed substantially higher 30-year event rates [35]. Persistently elevated hsCRP or IL-6 levels 3–12 months after ACS identify patients at greater risk of recurrence and mortality who may derive benefit from adjunctive inflammation-lowering therapy [1,3,5,7]. hsCRP has also been shown to identify cardiovascular risk even in individuals without standard modifiable risk factors (SMuRF-less). In the Women’s Health Study, women with hsCRP levels above 3 mg/L had a 77% higher risk of coronary heart disease, a 39% higher risk of stroke, and a 52% higher risk of total cardiovascular events over 30 years despite the absence of traditional risk factors, establishing inflammation as an independent driver of atherosclerotic risk [36].

Imaging further refines inflammatory risk assessment by localizing active vascular inflammation. ^18F-FDG PET identifies metabolically active plaques reflecting macrophage glycolytic flux, while ^18F-NaF PET highlights microcalcification associated with plaque healing and instability [37]. High-resolution MRI and OCT quantify fibrous-cap thickness, lipid-core burden, and cap disruption, aiding phenotype-specific risk stratification [25,26]. Integration of biomarker kinetics with imaging signatures may refine patient selection and optimal timing of anti-inflammatory therapy in post-ACS management.

Emerging strategies now target inflammation through multiple complementary pathways, including inhibition of P-selectin to limit platelet–leukocyte interactions, Bruton’s tyrosine kinase blockade to suppress NLRP3 activation, IL-6-JAK-STAT inhibition to blunt cytokine propagation, and 5-HT2B antagonism to modulate macrophage phenotype [3,17]. Combining mechanistic biomarkers with imaging-derived endotypes may enable precision-guided anti-inflammatory therapy to mitigate residual risk and improve outcomes after ACS.

## 3. Therapies for Secondary Prevention

### 3.1. Guideline Foundation: Lipids, Antithrombotics, and Residual Inflammatory Risk

Current ACC/AHA and ESC guidelines (Table 3) for secondary prevention after ACS emphasize high-intensity statin therapy, with ezetimibe and PCSK9 inhibitors as adjuncts for additional LDL-C lowering, and dual antiplatelet therapy for at least 12 months [1,2]. Statins exert both lipid-lowering and anti-inflammatory effects by inhibiting isoprenoid synthesis, reducing prenylation of small GTP-binding proteins such as Rho and Rac, and thereby attenuating NF-κB activation and lowering CRP and IL-6. Imaging studies have shown parallel reductions in plaque inflammation, necrotic core, and plaque volume independent of LDL-C changes [18,19]. Ezetimibe, PCSK9 inhibitors, and bempedoic acid provide incremental LDL-C reduction and modest hsCRP lowering (typically 10–20%), which is likely secondary to lipid lowering rather than a direct anti-inflammatory action [38,39,40]. These therapies reduce lipid burden and modestly reduce inflammatory cascades, yet many patients remain at high RIR.

Both societies endorse selective anti-inflammatory therapy for selected high-risk patients with RIR, typically hsCRP ≥ 2 mg/L, despite guideline-directed therapy [1,41]. However, hsCRP testing remains underutilized, and adoption of anti-inflammatory therapy is limited by uncertainty over thresholds, limited recommendations, and safety concerns. Mechanistically, RIR reflects persistent endothelial activation, immune cell infiltration, and maladaptive cytokine signaling despite aggressive lipid lowering, providing a rationale for integrating inflammation-targeted strategies into comprehensive secondary prevention.

### 3.2. Clinically Supported Therapies

Colchicine 

Colchicine is an inexpensive oral anti-inflammatory that inhibits microtubule polymerization, suppressing neutrophil activation, chemotaxis, and NLRP3 inflammasome signaling, thereby reducing IL-1β and IL-6 production and downregulating endothelial adhesion molecules and chemokines. Additional effects on platelet activation and endothelial dysfunction may contribute to antithrombotic benefit [42,43]. Efficacy for secondary prevention is supported by two pivotal trials. In COLCOT, 4745 patients with recent MI treated with colchicine at 0.5 mg daily for a median of 22.6 months had a 23% relative risk reduction in the composite of CV death, resuscitated cardiac arrest, MI, stroke, or urgent angina-driven revascularization (HR 0.77; 95% CI 0.61–0.96; *p* = 0.02), driven by fewer urgent revascularizations and strokes; benefit was greatest when initiated within 3 days post-MI (HR 0.52; 95% CI 0.32–0.84) [8,44]. In LoDoCo2, 5522 patients with stable CAD experienced a 31% relative risk reduction in CV death, MI, ischemic stroke, or ischemia-driven revascularization over 28.6 months (HR 0.69; 95% CI 0.57–0.83; *p* < 0.001), with a non-significant trend toward higher non-CV mortality (HR 1.51; 95% CI 0.99–2.31) [9]. Meta-analyses of up to nine RCTs (>30,000 participants) report a 12–25% reduction in major adverse CV events (HR 0.75; 95% CI 0.56–0.93), with consistent benefit across prior MI and stable CAD, which is comparable to, or exceeds, some adjunctive lipid-lowering therapies [45]. Benefit persists despite only modest hsCRP lowering, which supports downstream anti-inflammatory and antithrombotic effects [10].

Tolerability is generally favorable; myotoxicity risk is not increased at 0.5 mg daily in patients without severe renal/hepatic impairment or strong CYP3A4/P-gp inhibitors, although therapy is contraindicated when creatinine clearance is less than 15 mL/min or with severe hepatic dysfunction [46]. Mild gastrointestinal intolerance (mainly diarrhea) is the most frequent adverse effect, with no excess of serious infection, cancer, or non-CV death leading to discontinuation in 10% [45,46]. Discontinuation risk varies by study design: the relative risk of discontinuation was 1.60 overall, decreased to 1.34 after excluding non-placebo trials, and was lowest and nonsignificant (RR 1.26) in the three largest placebo-controlled studies. Importantly, the estimated net clinical benefit remained favorable, preventing 17.8 events per 1000 patients (*p* < 0.001), supporting colchicine as a high-value adjunct in secondary prevention [47].

In 2023, the FDA approved colchicine at 0.5 mg daily to reduce CV risk in adults with ASCVD or multiple risk factors, facilitating integration into secondary prevention [10,48]. ACC/AHA 2025 assigns a Class IIb (B-R) recommendation to consider colchicine in ACS and CCS to reduce MACE, and ESC 2023 gives it Class IIb (A) for long-term use in selected ACS patients and Class IIa (A) in CCS for secondary prevention when residual inflammatory risk or recurrent events are present [1,2]. Despite class recommendations, clinical evidence remains heterogeneous. Smaller ACS trials and pooled analyses show variable efficacy when colchicine is initiated late or in lower-risk populations. A recent network meta-analysis demonstrated that initiation beyond seven days post-MI yielded no significant reduction in recurrent ischemic events (IRR 0.94; 95% CI 0.81–1.09), whereas treatment within 24 h conferred greater benefit (IRR 0.72; 95% CI 0.58–0.89) [49]. The non-significant trend toward higher non-cardiovascular mortality observed in LoDoCo2 (HR 1.51; 95% CI 0.99–2.31) highlights the need for adequately powered confirmatory outcome trials to define optimal timing, safety, and patient selection for long-term anti-inflammatory therapy.

Canakinumab

Inflammation post-ACS is amplified by NLRP3 activation with IL-1β release and downstream IL-6 signaling. Canakinumab is a fully human monoclonal antibody that neutralizes IL-1β within the NLRP3 inflammasome pathway implicated in atherothrombosis [12,30]. In CANTOS, 10,061 patients with prior MI and hsCRP ≥ 2 mg/L were randomized to canakinumab 50, 150, or 300 mg subcutaneously every 3 months versus placebo. Over 3.7 years, the 150 mg dose reduced nonfatal MI, nonfatal stroke, or cardiovascular death by 15% (HR 0.85; 95% CI 0.74–0.98; *p* = 0.021) without affecting lipids, corresponding to an absolute risk reduction of about 0.6% per year and an NNT of 167. Higher doses did not add efficacy or worsen side effects [12]. Benefit correlated with on-treatment reductions in hsCRP and IL-6, with the greatest effect in patients achieving hsCRP < 2 mg/L (HR 0.75; 95% CI 0.66–0.85; *p* < 0.0001 for MACE; cardiovascular mortality HR 0.69; 95% CI 0.56–0.85) [50]. However, canakinumab increased fatal infection and sepsis (HR 1.48) and did not reduce all-cause or CV mortality (HR 0.94; 95% CI 0.83–1.06) [12].

Despite demonstrating proof-of-concept of cytokine-targeted therapy, its clinical applicability is limited by safety concerns, lack of regulatory approval, and an annual cost of nearly USD 200,000, with cost-effectiveness analyses remaining unfavorable even with modeled 90% price reductions [51,52]. Current use is largely confined to rare autoinflammatory syndromes.

### 3.3. Investigational Therapies

IL-1/IL-6 Axis (Anakinra, Ziltivekimab, and Tocilizumab)

Anakinra, a recombinant IL-1 receptor antagonist, has been evaluated in multiple STEMI trials. In VCU ART3, 100 mg subcutaneously once or twice daily for 14 days reduced hsCRP AUC from 214 to 67 mg·day/L (*p* < 0.001) without improving LV remodeling or LVEF at 12 months, but importantly, it lowered death or new HF (9.4% vs. 25.7%, *p* = 0.046) and eliminated HF hospitalizations (0% vs. 11.4%, *p* = 0.011) [53]. Pooled VCU ART data showed that there was no effect on recurrent ischemic events (HR 1.08; 95% CI 0.52–2.24) but a marked reduction in death or HF (HR 0.16; 95% CI 0.04–0.65), with greater benefit in patients with a high inflammatory burden [54]. No excess serious infections were reported, though mild side-effects of injection-site reactions were more frequent. A network meta-analysis of 23 RCTs with 28,220 patients suggested that initiation within 24 h may reduce HF events (IRR 0.38; 95% CI 0.18–0.79) but certainty is low, and a 2024 Cochrane review rated evidence for MACE reduction as low to very low [49,55]. Anakinra remains investigational with no ASCVD prevention approval.

Ziltivekimab, a fully human monoclonal antibody targeting the IL-6 ligand, achieved dose-dependent hsCRP reductions of 77%, 88%, and 92% at 7.5, 15, and 30 mg monthly in RESCUE versus 4% with placebo, and it also lowered fibrinogen, SAA, haptoglobin, secretory phospholipase A_2_, and Lp(a) [15,56]. Secondary analyses showed reduced neutrophil-to-lymphocyte ratio, supporting broad anti-inflammatory effects [57]. Tolerability was favorable, with no serious cytopenias. A phase 3 ZEUS outcomes trial in approximately 6200 patients with ASCVD, CKD, and elevated hsCRP is ongoing; ziltivekimab is not yet approved [58]. Additionally, Tocilizumab, a monoclonal IL-6 receptor antagonist, has been studied in acute STEMI. In ASSAIL-MI, a single 280 mg IV infusion within 6 h of symptom onset increased myocardial salvage index by 5.6 percentage points (69.3% vs. 63.6%; *p* = 0.04) and reduced microvascular obstruction but did not significantly reduce final infarct size or short-term MACE [59]. While it improves vascular function in other contexts, its effect on cardiac outcomes remains unproven.

Overall, the IL-1/IL-6 inhibition remains biologically compelling post-ACS, with canakinumab providing the proof-of-concept. Anakinra shows potential for HF prevention without ischemic benefit, ziltivekimab yields profound biomarker reductions with outcomes pending, and tocilizumab provides mechanistic benefit without clear clinical impact. Guideline adoption awaits phase 3 results and likely applies to patients with high baseline inflammatory burden. Cost-effectiveness post-ACS is unknown, and pricing and access barriers will likely mirror other biologics.

NLRP3 Inflammasome and P-Selectin Inhibition (Dapansutrile and Inclacumab)

Dapansutrile is an oral, selective NLRP3 inflammasome inhibitor that acts upstream of IL-1β production [60]. Phase 2 studies in acute gout and other inflammatory conditions have shown significant hsCRP reductions and favorable safety at doses up to 1000 mg/day [60,61]. The oral route offers practical advantages for long-term therapy compared with injectable biologics. However, no clinical outcomes data exist for post-ACS prevention. Its anticipated effects on IL-1β, IL-6, and hsCRP mirror those of canakinumab and anakinra, but efficacy and safety remain unproven in large-scale trials [62]. Furthermore, inclacumab is a fully human monoclonal antibody targeting P-selectin, and it is hypothesized to attenuate platelet–leukocyte interactions and downstream vascular inflammation [21]. In SELECT-ACS, 530 NSTEMI patients undergoing PCI received a single pre-procedural infusion of inclacumab (5 or 20 mg/kg) or placebo. The 20 mg/kg dose within 3 h before PCI reduced periprocedural injury, lowering troponin I by 24.4% (*p* = 0.05) and CK-MB by 27.3% (*p* = 0.057) at 24 h, with a larger effect when given <180 min before PCI (troponin I—43.5%, *p* = 0.019) [22]. The trial was not powered for long-term MACE, and effects on recurrent MI, HF, or mortality are unknown. Safety was comparable to placebo. Both agents remain investigational and are not approved for secondary prevention of ASCVD.

### 3.4. Neutral or Negative Targets (Methotrexate, sPLA_2_, Lp-PLA_2_, p38 MAPK, and Complement C5i)

Several anti-inflammatory agents have failed to demonstrate cardiovascular benefit in large trials, highlighting the need for biologically validated targets and appropriate patient selection. Methotrexate, despite the established anti-inflammatory effects in rheumatologic disease largely mediated through adenosine release, neither reduced IL-1β, IL-6, or hsCRP nor lowered MACE in the CIRT trial of 4786 patients with stable ASCVD and diabetes or metabolic syndrome (HR 0.96; 95% CI 0.79–1.16), potentially reflecting its limited activity on the NLRP3-IL-1β-IL-6 axis and inclusion of participants without elevated baseline inflammation [63]. Varespladib, a secretory phospholipase A_2_ inhibitor, was terminated early in VISTA-16 for futility and increased MI risk (HR 1.66; 95% CI 1.16–2.39) [64]. Additionally, darapladib (lipoprotein-associated phospholipase A_2_ inhibitor) showed no benefit in SOLID-TIMI 52 (HR 0.94; 95% CI 0.85–1.03), and losmapimod (p38 MAPK inhibitor) was neutral in LATITUDE-TIMI 60 (HR 1.16; 95% CI 0.91–1.47) [65,66]. Pexelizumab, a complement C5 inhibitor, also failed in STEMI and CABG populations, though exploratory analyses suggested possible mortality benefit in high-risk surgical subgroups [67,68]. These findings emphasize the need to target pathways with causal links to atherothrombosis and to select patients with demonstrable RIR.

## 4. Adjunct Cardiometabolic Modulators (GLP-1 RAs and SGLT2i)

GLP-1 RAs and SGLT2i are cornerstone cardiometabolic therapies with complementary metabolic, vascular, and anti-inflammatory effects for secondary prevention in ASCVD [13,14]. GLP-1 RAs produce weight loss, improve glycemic and lipid control, lower blood pressure, and reduce inflammatory signaling [69,70]. They also lower CRP, IL-6, TNF-α, and monocyte–macrophage activation, improve endothelial function, and attenuate vascular inflammation [71,72,73]. In SELECT, semaglutide 2.4 mg weekly reduced CV death, nonfatal MI, or stroke by 20% in 17,604 overweight/obese adults without diabetes (HR 0.80; 95% CI 0.72–0.90; *p* < 0.001), with NNTs of 67 for MACE and 207 for MI. Meta-analyses (>100,000 patients) confirm reductions in MACE (RR 0.87; 95% CI 0.81–0.93), MI (RR 0.86; 95% CI 0.79–0.94), stroke (RR 0.88; 95% CI 0.81–0.96), and CV death (RR 0.87; 95% CI 0.80–0.95) [74,75]. SUSTAIN-6 and REWIND reported 26% and 12% MACE reductions (HR 0.74; 95% CI 0.58–0.95 and HR 0.88; 95% CI 0.79–0.99, respectively), and LEADER showed liraglutide lowered MACE (HR 0.87; 95% CI 0.78–0.97) and all-cause mortality (HR 0.85; 95% CI 0.74–0.97) [76,77,78]. Benefits were consistent across subgroups, including prior MI or stroke, and reflect both metabolic and direct anti-inflammatory actions [79]. Post-MI data suggest improved survival and fewer recurrences, particularly in higher BMI or PCI populations, with meta-analyses showing a 14% MI risk reduction [74,80,81]. Adverse effects are mainly gastrointestinal; hypoglycemia risk is low without insulin or sulfonylureas [13,74]. The guideline-recommended CV dose is semaglutide 2.4 mg weekly with gradual titration [1].

SGLT2i benefits via hemodynamic unloading improved myocardial energetics, antifibrotic effects, and sympathetic inhibition [69,70]. Anti-inflammatory effects have shown to lower CRP, ferritin, leptin, and plasminogen activator inhibitor-1, increase adiponectin, improve vascular function, and attenuate cardiac remodeling [69,82]. Empagliflozin and dapagliflozin been have shown to reduce MACE in type 2 diabetes with ASCVD (HR 0.89; 95% CI 0.83–0.96), with stronger effects on HF hospitalization (HR 0.69; 95% CI 0.61–0.79) and kidney disease progression (HR 0.55; 95% CI 0.48–0.63) [83]. EMPA-REG OUTCOME established empagliflozin’s benefit, showing large reductions in cardiovascular death (HR 0.62; 95% CI 0.49–0.77), heart failure hospitalization (HR 0.65; 95% CI 0.50–0.85), and all-cause mortality (HR 0.68; 95% CI 0.57–0.82) [84]. Post-MI trials (EMPACT-MI, DAPA-MI) were neutral for CV death/HF hospitalization in patients without diabetes or HF, but pooled analyses suggest early initiation reduces MACE and HF hospitalization [85,86]. Observational post-AMI studies in diabetes further support these findings, with lower cardiovascular death (HR 0.42; 95% CI 0.28–0.63), heart failure hospitalization (HR 0.59; 95% CI 0.47–0.74), and all-cause mortality (HR 0.69; 95% CI 0.56–0.85) without more recurrent MI or stroke [87]. Adverse events include genital infections, hypovolemia, and rare ketoacidosis; the guideline-recommended dose is 10 mg daily [1,14].

Together, these classes are complementary, with combination therapy offering potential maximal protection in appropriately selected patients [88]. Observational data suggest additive benefits with combination therapy, including a 30% MACE reduction and a 57% reduction in serious renal events versus GLP-1 RA alone, with good tolerability and outcomes similar to SGLT2i [89,90]. Cost-effectiveness generally favors SGLT2i and colchicine, while GLP-1 RAs remain high-value in selected high-risk patients [91]. Guidelines recommend GLP-1 RAs and SGLT2i for secondary prevention in ASCVD, including post-MI and in overweight or obese patients with stable disease. Early post-ACS initiation appears safe and may accelerate benefit, although stable-phase initiation yields comparable long-term outcomes [1,2,41,92].

## 5. Emerging Mechanisms and Platforms

### 5.1. Immune-Signaling Modulators (BTK, JAK/STAT, and 5-HT_2_B)

Evidence for novel mechanistic therapies in secondary prevention post-ACS/MI remains largely confined to preclinical studies [93,94]. Triptolidiol, a triptolide derivative with anti-inflammatory properties, has demonstrated favorable effects in translation research, with no human cardiovascular data [93]. Dasatinib, a BCR-ABL1 inhibitor approved for hematologic malignancies, has shown preclinical evidence of reduced cholesterol uptake, suggesting potential anti-atherosclerotic effects. However, in oncology cohorts, dasatinib was associated with a 2–4% incidence of cardiovascular ischemic events, though these rates were not elevated compared with matched cohorts [95,96].

BTK inhibitors such as ibrutinib, acalabrutinib, and zanubrutinib are widely used in B-cell malignancies. BTK mediates B-cell receptor signaling and participates in innate immune activation through NLRP3 inflammasome assembly; its inhibition potentially reduces IL-1β production and downstream cytokine cascades implicated in plaque destabilization [97]. While preclinical data suggest immune modulation may influence atherosclerosis, clinical use has been associated with atrial fibrillation, bleeding, and hypertension, and pooled analyses have not demonstrated cardiovascular benefit compared with standard therapy [97,98].

Similarly, JAK/STAT inhibitors such as tofacitinib and ruxolitinib target cytokine-driven pathways central to vascular inflammation. IL-6-JAK-STAT signaling drives hepatic CRP synthesis and systemic inflammation, while IFN-γ-JAK-STAT promotes macrophage activation and plaque vulnerability [99,100]. Although these agents have shown potential to reduce cardiovascular events in autoimmune and myeloproliferative disorders, no randomized trials have evaluated their use after ACS or MI due to concerns regarding prothrombotic risk and cardiovascular safety [99,100,101,102]. Interest has also extended to 5-HT_2_B receptor modulators, which reduce fibrosis and adverse remodeling in animal models of MI, though they remain untested in clinical populations [103,104]. These therapies remain exploratory in the absence of efficacy and safety data, and current translational focus continues to emphasize established agents with outcome trial evidence for secondary prevention.

### 5.2. Advanced Platforms (Nanozymes, CRISPR)

Nanozymes are engineered nanomaterials with intrinsic enzyme-mimetic activity, including superoxide dismutase, catalase, and glutathione peroxidase, that scavenge ROS in ischemia–reperfusion injury, plaque destabilization, and adverse remodeling after ACS. By restoring redox balance, nanozymes suppress NF-κB signaling, limit inflammatory cell recruitment, and promote reparative macrophage polarization. Preclinical rodent and porcine studies demonstrate robust cardioprotection, with Fe-Cur@TA nanozymes achieving a tenfold increase in myocardial retention, reducing infarct size, improving LVEF by more than 10 percentage points, and attenuating cytokine release without toxicity [105,106]. Other platforms, including Prussian blue, PtIr bimetallic, and MnO_2_ nanozymes, have shown multi-enzyme activity, 30 to 40 percent reductions in infarct size and fibrosis, and improved microvascular density [107,108,109]. Ex vivo human cell studies confirm anti-inflammatory effects with more than 50 percent reductions in TNF-α, IL-1β, and NF-κB expression and minimal cytotoxicity, while large-animal safety assessments indicate favorable biocompatibility, although data on chronic exposure and drug–drug interactions remain limited [106]. No human clinical trials have yet been conducted. CRISPR-based therapeutics represent a fundamentally different approach, enabling permanent modification of pro-inflammatory or pro-remodeling genes such as TLR4, IL-1β, and CaMKIIδ. In preclinical models, CRISPR-mediated disruption of these targets reduces cytokine production, preserves myocardial function, and limits infarct expansion, with similar effects observed in ex vivo human cell editing [110,111,112]. Current cardiovascular CRISPR trials focus largely on lipid modulation, such as PCSK9 editing, rather than post-infarct inflammation, reflecting major translational barriers including targeted cardiac delivery, avoidance of off-target edits, immunogenicity of Cas proteins, and stringent regulatory thresholds for chronic, non-lethal conditions [113,114]. While nanozymes appear closer to clinical readiness, given their rapid, reversible pharmacologic effects and emerging safety data, CRISPR remains an early-stage platform requiring significant advances in delivery and safety before entering secondary prevention trials.

## 6. Discussion

Inflammation is central to ACS, orchestrating plaque progression, destabilization, rupture, and acute events. The interplay of innate and adaptive immunity, cytokines, chemokines, and pattern-recognition receptors drives both acute injury and long-term remodeling [1,2,3]. RIR has emerged as a modifiable determinant of recurrence, with biomarkers such as hsCRP, IL-6, leukocyte counts, and composite indices providing independent prognostic value. An early IL-6 surge followed by an hsCRP peak at about 48 h, especially if sustained after discharge, defines a high-risk phenotype [4,5,27].

Guidelines emphasize rapid initiation of therapy, highlighting statins as the foundation for combining lipid-lowering with pleiotropic anti-inflammatory effects. Low-dose colchicine at 0.5 mg daily offers the most proven adjunct, particularly for patients with hsCRP ≥ 2 mg/L or high-risk comorbidities. Colchicine’s benefits, supported by consistent outcome reductions in COLCOT and LoDoCo2, likely extend beyond modest biomarker changes, reflecting effects on neutrophil trafficking, NLRP3 signaling, endothelial activation, and platelet–leukocyte interactions [8,9,10]. Cardiometabolic agents, including GLP-1 receptor agonists and SGLT2 inhibitors, further reduce risk through overlapping metabolic and inflammatory pathways, strengthening their role in post-ACS care [13,75,84,115].

Targeting the cytokine axis strengthens causal inference but requires careful selection. In CANTOS, IL-1β blockade reduced recurrent events in proportion to IL-6 and hsCRP suppression, though safety and cost limited uptake [12,50]. Anakinra attenuates systemic inflammation with signals for heart failure prevention, whereas ziltivekimab drives marked reductions in hsCRP and IL-6 and is advancing in phase 3 [53,57]. Upstream approaches, including oral NLRP3 inhibition with dapansutrile and peri-procedural P-selectin antagonism with inclacumab, broaden the pipeline, though definitive outcome data remain pending [11,21].

Other strategies have failed in coronary disease, including methotrexate, lipoprotein-associated phospholipase A_2_ inhibitors, p38 MAPK inhibitors, and complement blockades, likely reflecting off-target effects or unselected populations. By contrast, therapies that suppress the IL-1 and IL-6 pathway in biomarker-enriched cohorts consistently reduce events [12,15,56].

Next-generation approaches remain preclinical. Nanozymes and cardiac-targeted platforms reduce oxidative stress and inflammation in animal models [106,116,117]. CRISPR-based modulation of TLR4 or CaMKIIδ is feasible ex vivo but is limited by in vivo delivery and safety. Parallel efforts explore NET inhibition, MPO blockades, and restoration of specialized pro-resolving mediators.

Practical hurdles remain, and real-world adoption is slowed by underuse of hsCRP and IL-6 testing, uncertain thresholds and duration of therapy, polypharmacy, and insurance barriers. Simple solutions may include routine hsCRP measurement at 4 to 12 weeks post-ACS, EHR-embedded prompts for colchicine initiation at hsCRP ≥ 2 mg/L, pharmacist-led screening, and shared decision tools emphasizing absolute risk reduction. Practical implementation must also consider timing; similar to lipid-lowering strategies, where early initiation improves outcomes, anti-inflammatory therapy may require ultra-early initiation to maximize benefit, yet this remains untested in large trials [118]. For biologics, infection risk and cost currently outweigh benefit, with cost-effectiveness achieved only when targeted at extreme residual inflammatory risk.

## 7. Evidence Gaps and Future Directions

Despite advances in lipid-lowering and antithrombotic therapy, major gaps persist in the timing, duration, and personalization of inflammation- and metabolism-targeted strategies after ACS. Most randomized trials, including CANTOS, COLCOT, and LoDoCo2, initiated therapy days to weeks after the index event, well beyond the 3-to-7-day peak of inflammatory activity when NLRP3 activation, NETosis, endothelial dysfunction, and defective resolution are most modifiable [3,4,119]. A recent network meta-analysis of 23 randomized trials including 28,220 patients demonstrated that both colchicine and anakinra reduced major adverse cardiovascular events and heart failure events, respectively, but these benefits were observed only when therapy was initiated within 24 h of symptom onset [49]. No adequately powered trial has prospectively tested ultra-early initiation within 24 h in biomarker-selected ACS patients. Ongoing studies such as COCOMO-ACS (early colchicine guided by OCT-defined plaque phenotype) and ZEUS (ziltivekimab in high-risk CKD/ASCVD) aim to address this gap. Their rationale parallels contemporary lipid management, where prompt initiation of high-intensity statins and early PCSK9 inhibition lower LDL-C, attenuate vascular inflammation, and promote plaque stabilization [118]. Collectively, these strategies signal a convergent therapeutic paradigm that integrates lipid and immune modulation, emphasizing ultra-early intervention during the transient post-ACS inflammatory window when risk and therapeutic responsiveness are greatest.

The optimal duration of anti-inflammatory therapy after ACS remains uncertain. Canakinumab provided sustained benefit over 3.7 years in CANTOS but raised infection concerns. Post hoc COLCOT data suggest the benefit may wane after colchicine discontinuation, yet no trial has tested structured de-escalation or biomarker-guided withdrawal [45,120]. A treat-to-target approach remains untested. Although hsCRP and IL 6 identify residual inflammatory risk, thresholds, timing, and monitoring strategies are unclear [121,122].

Comorbidities shape inflammatory phenotypes and modulate therapeutic response. Diabetes promotes neutrophil-dominant inflammation and NETosis [88,123]. Chronic kidney disease amplifies IL-6-driven risk and increases susceptibility to ischemic and bleeding complications [124,125]. Autoimmune disease and frailty add further complexity, yet these high-risk groups remain underrepresented in trials [126,127].

Pooling heterogeneous ACS phenotypes obscures mechanistic insight. Rupture, erosion, calcified nodules, MINOCA, and SCAD differ in biology and therapeutic implications [28]. OCT-defined plaque erosion can be managed without stenting using intensive antithrombotic therapy, and potential benefit from anti-inflammatory therapy remains plausible but unproven [128,129]. MINOCA and SCAD, which disproportionately affect women, are still excluded from most anti-inflammatory outcome trials despite clear inflammatory components [7]. No trial has prospectively stratified therapy by mechanism. Ongoing studies such as COCOMO ACS are testing early colchicine with imaging-defined phenotypes [130]. Precision imaging tools such as FDG PET, OCT, and molecular profiling, including CHIP and NETosis high states, may enable phenotype-specific risk assessment and guide therapy selection but remain investigational [93,131].

Representation in cardiovascular trials must improve. Women comprise only 20–25 percent of trial cohorts despite sex-specific immune and plaque biology, while young patients, minorities, and those with obesity remain underrepresented [132]. Endpoints should expand beyond recurrent MI to include heart failure, atrial fibrillation, peripheral artery disease progression, microvascular dysfunction, renal decline, quality of life, and functional recovery. CANTOS suggested reductions in heart failure hospitalization, and anakinra lowered NT proBNP in high CRP ACS, supporting broader endpoints [50,54,133].

Combination or sequencing strategies such as colchicine plus GLP 1 receptor agonists or SGLT2 inhibitors are supported by observational data suggesting additive benefit, but no randomized controlled trials have directly tested these approaches in post-ACS care. Real-world data indicate that uptake of GLP 1 receptor agonists and SGLT2 inhibitors after ACS remains suboptimal, with only a minority of eligible patients receiving both agents and even fewer receiving colchicine in addition [90,134]. Barriers include lack of direct evidence, cost, access, polypharmacy, and uncertainty in patient selection and sequencing [88,134]

Priorities for the next generation of trials include ultra-early initiation, biomarker-guided and imaging-based precision strategies, inclusion of diverse high-risk populations, and integration of metabolic, thrombotic, and inflammatory modulation. Programs such as ZEUS for ziltivekimab, SELECT for semaglutide, and COCOMO ACS for colchicine with mechanistic imaging are addressing key aspects, while SUMMIT extends the multidomain model to obesity-related HFpEF. Pragmatic implementation models, AI-driven phenotyping, and equity-focused trial design will be essential to translate these advances into routine care. Aligning inflammation-targeted therapy with precision medicine offers a credible path to reduce residual risk and improve long-term outcomes in ACS.

## 8. Conclusions

Residual inflammatory risk persists after ACS despite optimized lipid-lowering and antithrombotic therapy. Current evidence supports low-dose colchicine as an adjunct for secondary prevention, whereas cytokine- and inflammasome-targeted approaches remain investigational pending definitive phase 3 trials and clearer safety and cost profiles (summary Table 4). The field remains in the early stages of defining the optimal anti-inflammatory strategy, including agent selection, timing and duration, and biomarker-guided treat-to-target using hsCRP and IL-6, with attention to phenotype and comorbidity. Priorities include adequately powered, mechanism-aware trials, pragmatic pathways for equitable implementation, and integration with cardiometabolic therapies.

## Figures and Tables

**Table 1:** Chemokine-Receptor and Adhesion Axes in Coronary Atheroinflammation: Key leukocyte–endothelial signaling pathways mediating monocyte and neutrophil recruitment, adhesion, and transmigration in atherosclerotic plaque inflammation, with translational correlates from experimental and clinical studies.

Axis	Ligand (Source)	Receptor (Leukocyte)	Principal Role	Translational Notes
CCL2-CCR2	Endothelium, SMCs, macrophages	CCR2 on monocytes	Major driver of classical monocyte entry	LDL-C lowering reduces CCR2-dependent mobilization [18,19]
CX3CL1-CX3CR1	Endothelium, SMCs	CX3CR1 on monocytes	Adhesion and chemotaxis under shear	Targeting reduces macrophage persistence and inflammatory cell retention [3,17]
CCL5-CCR5	Platelets, T cells	CCR5 on monocytes, T cells	Recruitment to plaque shoulders	CCR5 antagonism limits leukocyte influx and plaque inflammation [3]
CXCL1-CXCR2	Endothelium, platelets	CXCR2 on neutrophils	Neutrophil recruitment and NETosis	CXCR2 blockade reduces neutrophil trafficking; P-selectin inhibition under study [20]
P-selectin-PSGL-1	Platelets, endothelium	PSGL-1 on leukocytes	Rolling and platelet–leukocyte aggregation	Inclacumab reduced peri-PCI injury in SELECT-ACS [21,22]
VLA-4-VCAM-1	Endothelium	VLA-4 on monocytes	Firm adhesion and transmigration	VCAM-1 expression decreases with lipid-lowering and statin therapy [16,17]
LFA-1-ICAM-1	Endothelium	LFA-1 on leukocytes	Firm adhesion and diapedesis	Broad anti-inflammatory and lipid-lowering therapies downregulate ICAM-1 [16]

**Table 2:** Inflammation-related biomarkers post-ACS: Inflammation-related biomarkers provide mechanistic and prognostic insights beyond conventional risk markers. hsCRP and IL-6 are validated for residual inflammatory risk, and NT-proBNP and GDF-15 add prognostic utility, while others (e.g., MPO, GlycA, suPAR, CHIP, and NET markers) remain investigational. Current guidelines suggest hsCRP (≥2 mg/L) and NT-proBNP for post-ACS risk stratification.

Biomarker	Pathophysiology	Prognostic Value	Clinical Utility
hsCRP	Acute-phase reactant; IL-1β/IL-6 axis	Predicts recurrent MI, MACE, and mortality	Validated threshold ≥ 2 mg/L; trial enrichment
IL-6	Upstream cytokine driving CRP	Early rise predicts short- and long-term MACE, mortality	Prognostic; investigational for therapy guidance
Leukocyte count/NLR/MLR	Innate immune activation	Higher values predict near-/long-term mortality	Widely available; not guideline-mandated
Fibrinogen	Acute-phase, prothrombotic	Linked to recurrent events, mortality	Available; not guideline-endorsed
Serum amyloid A	Acute-phase; plaque instability	Predicts adverse outcomes	Emerging; not routine
GDF-15	Stress/inflammatory marker (TGF-β)	Strong predictor of CV death, MI	Prognostic; limited integration
NT-proBNP	Wall stress/remodeling	Predicts HF, death, MACE	HF guidelines; prognostic in ACS
Composite indices (TyG, TG/HDL-C, PIV)	Metabolic-immune integration	Additive to cTnI, STEMI risk	Observational; investigational
Lp(a)	Pro-atherogenic; IL-6-linked	Residual risk post-ACS	Measured once in adulthood; not inflammation-specific
MPO	NETosis, oxidative stress	Linked to erosion, adverse outcomes	Mechanistic; investigational
CHIP	Clonal hematopoiesis → IL-1β/IL-6	Higher recurrent-event risk	Investigational
MicroRNAs (e.g., miR-146a, -155)	Post-transcriptional immune regulation	Experimental predictors	Preclinical
GlycA	Systemic inflammation (NMR signal)	Independent ACS/ASCVD risk	Emerging; not guideline-endorsed
suPAR	Chronic immune activation	Associated with MACE, renal decline	Emerging; not guideline-endorsed
NET markers (cfDNA, MPO-DNA)	Direct NETosis indices	Linked to no-reflow, outcomes	Investigational
Lp-PLA_2_	Lipoprotein-associated enzyme	Prognostic signals reported	Not recommended; darapladib neutral
sST2	Fibrosis/inflammation	Prognostic for HF post-MI	Prognostic; not ACS guideline-mandated

**Table 3:** Guideline Recommendations for Adjunctive Therapies in Secondary Prevention Post-ACS/CCS: Summary of ACC/AHA and ESC recommendations for adjunctive therapies beyond statins. Both societies endorse high-intensity statins with nonstatin therapies (ezetimibe and PCSK9 inhibitors) if LDL-C goals are unmet. Colchicine is assigned a IIb recommendation in ACS, with stronger support in coronary syndromes when residual inflammatory risk persists. GLP-1 receptor agonists and SGLT2 inhibitors are recommended in ASCVD with type 2 diabetes, with expanding indications for obesity, HF, and CKD. hsCRP is recognized as a marker of residual inflammatory risk, with ESC providing a IIa recommendation for biomarker-guided therapy selection.

Therapy	ACC/AHA	ESC
Statins	Class I, LOE A. High-intensity statin for all ACS; LDL-C goal < 70 mg/dL. If goal not reached, add ezetimibe ± PCSK9 inhibitor.	Class I, LOE A. High-intensity statin for all ACS; LDL-C goal < 55 mg/dL and ≥50% reduction. If goal not reached, add ezetimibe ± PCSK9 inhibitor.
Colchicine	Class IIb, LOE B-R. May be considered in ACS and CCS to reduce MACE in selected patients.	ACS: Class IIb, LOE A. May be considered long-term in selected patients. CCS: Class IIa, LOE A. Should be considered for secondary prevention when residual inflammatory risk or recurrent events are present.
GLP-1 receptor agonists	ACS: No specific COR for early initiation. May be started at discharge when otherwise indicated; semaglutide 2.4 mg lowers MACE in SELECT but not tested early post-ACS. CCS: Class I, LOE A in ASCVD with T2D to reduce MACE; Class IIa, LOE B-R for overweight/obesity requiring pharmacotherapy (semaglutide preferred).	CCS: Class I, LOE A in ASCVD with T2D to reduce CV events; Class IIa, LOE B semaglutide may be considered in overweight/obese patients without T2D to reduce CV death/MI/stroke.
SGLT2 inhibitors	CCS: Class I, LOE A in ASCVD with T2D to reduce MACE; Class IIa, LOE B-R in HF with LVEF > 40% to reduce HF hospitalization and improve quality of life. ACS: No specific COR; do not defer initiation at discharge when indicated for T2D, HF, or CKD.	CCS/HF/CKD: Class I, LOE A in ASCVD with T2D to reduce CV events; strongly recommended across HF and CKD populations.
Biomarker-guided therapy	hsCRP may inform risk stratification and identify residual inflammatory risk. Not a routine Class I/II recommendation for initiation or titration.	Class IIa, LOE B. hsCRP ≥ 2 mg/L identifies residual inflammatory risk and may support selection of anti-inflammatory therapy in secondary prevention.

**Table 4:** Anti-inflammatory therapies in secondary prevention after ACS: Summary of agents targeting inflammation and metabolism in post-ACS care. Colchicine is FDA-approved for ASCVD; canakinumab, anakinra, tocilizumab, and ziltivekimab demonstrate biologic activity but lack regulatory approval. GLP-1 receptor agonists and SGLT2 inhibitors provide cardiometabolic benefit and are guideline-endorsed in ASCVD with type 2 diabetes, with ongoing trials post-ACS. Prior agents, including varespladib, darapladib, losmapimod, and pexelizumab, were neutral or harmful. Emerging strategies, including oral NLRP3 inhibition, P-selectin blockade, JAK/STAT and BTK inhibitors, CRISPR-based therapies, and nanozymes, remain exploratory and unproven in outcomes studies.

Drug/Platform	Mechanism	Key Trials	Effects	Safety	Regulatory Status
Canakinumab	IL-1β monoclonal antibody	CANTOS	↓ MACE at 150 mg (HR = 0.85); no mortality benefit	↑ fatal infections/sepsis	Not approved for CV risk reduction
Anakinra	IL-1 receptor antagonist	VCU-ART pooled	↓ hsCRP; ↓ HF events at 1 yr; no ischemic benefit	Injection-site reactions; no increase in serious infections vs. placebo	Not approved
Tocilizumab	IL-6 receptor antagonist	ASSAIL-MI	↑ myocardial salvage index; no significant infarct-size or outcome difference	Well tolerated; no excess AEs	Not approved
Ziltivekimab	IL-6 ligand monoclonal antibody	RESCUE; ZEUS (ongoing)	Large, dose-dependent ↓ hsCRP (60–90%); ↓ neutrophil-to-lymphocyte ratio	Well tolerated in phase 2	Investigational
Colchicine	Microtubule inhibitor; NLRP3 blockade	COLCOT; LoDoCo2	↓ ischemic events (HR 0.77–0.69); ↓ stroke and revascularization	GI intolerance; small ↑ pneumonia signal; rare myotoxicity	FDA-approved (0.5 mg/d to reduce CV events in adults with ASCVD or at risk)
Dapansutrile	Oral NLRP3 inflammasome inhibitor	Phase 2 (gout, HF)	↓ hsCRP in gout; no ASCVD outcomes yet	Generally well tolerated in early trials	Investigational
Inclacumab	Anti-P-selectin monoclonal antibody	SELECT-ACS	↓ troponin I/CK-MB when infused pre-PCI; no outcomes data	Well tolerated	Investigational
Varespladib	sPLA2 inhibitor	VISTA-16	↑ MI risk; trial terminated for futility/possible harm	Unfavorable safety profile	Development terminated for ACS
Darapladib	Lp-PLA2 inhibitor	STABILITY; SOLID-TIMI 52	Neutral on MACE; no benefit	Diarrhea; odor complaints	Not approved
Losmapimod	p38 MAPK inhibitor	LATITUDE-TIMI 60	Neutral; no reduction in CV events	Acceptable safety; trial stopped for futility	Not approved
Pexelizumab	Complement C5 inhibitor	APEX-AMI; PRIMO-CABG II	Neutral overall; exploratory mortality signals not confirmed	Acceptable safety	Not approved
GLP-1 RAs	GLP-1 receptor agonist	SELECT; LEADER; SUSTAIN-6; REWIND	↓ MACE (SELECT HR = 0.80 in overweight/obesity without diabetes; class benefit in T2D)	GI intolerance; low hypoglycemia risk	FDA-approved (semaglutide 2.4 mg to reduce CV events in adults with CVD + overweight/obesity)
SGLT2 inhibitors	SGLT2 inhibition	EMPA-REG OUTCOME; EMPACT-MI; DAPA-MI	In T2D + ASCVD: ↓ CV death and HF hospitalization; post-MI: EMPACT-MI neutral on primary; DAPA-MI improved cardiometabolic composite, no MACE	Genital infections; rare ketoacidosis	FDA-approved
Methotrexate	Non-specific anti-inflammatory	CIRT	No reduction in inflammation or events	↑ LFTs, cytopenias, non-basal-cell skin cancers	Not approved
JAK inhibitors (e.g., tofacitinib, ruxolitinib)	JAK/STAT pathway inhibition	ORAL Surveillance (safety)	No ASCVD outcomes; ↑ MACE/VTE/malignancy/death vs. TNF-i in RA	Boxed warning for CV risk	Not approved
BTK inhibitors (e.g., ibrutinib, acalabrutinib, zanubrutinib)	BTK inhibition	None (oncology only)	No CV benefit	↑ atrial fibrillation, bleeding, hypertension (oncology experience)	Not approved
CRISPR-based therapeutics	Gene editing of inflammatory/cardiac targets (e.g., TLR4, IL-1β, CaMKIIδ)	Preclinical	↓ cytokines; preserved cardiac function in animal models	Delivery/off-target risks; immunogenicity	Preclinical
Nanozyme platforms	ROS scavenging via enzyme-mimetic nanomaterials	Preclinical	↓ infarct size; ↑ LVEF; ↓ TNF-α/IL-1β in animal MI models	Favorable in animals; human safety unknown	Preclinical

↑ = Increase; ↓ = Decrease.

## Data Availability

No new data were created or analyzed in this study. Data sharing is not applicable to this article as it is based entirely on previously published literature.
